# Krill Oil Treatment Increases Distinct PUFAs and Oxylipins in Adipose Tissue and Liver and Attenuates Obesity-Associated Inflammation via Direct and Indirect Mechanisms

**DOI:** 10.3390/nu13082836

**Published:** 2021-08-18

**Authors:** Eveline Gart, Kanita Salic, Martine C. Morrison, Martien Caspers, Wim van Duyvenvoorde, Marieke Heijnk, Martin Giera, Ivana Bobeldijk-Pastorova, Jaap Keijer, Andreas B. Storsve, Petter-Arnt Hals, Robert Kleemann

**Affiliations:** 1 Department of Metabolic Health Research, The Netherlands Organisation for Applied Scientific Research (TNO), 2333 CK Leiden, The Netherlands; ekzs@novonordisk.com (K.S.); martine.morrison@tno.nl (M.C.M.); wim.vanduyvenvoorde@tno.nl (W.v.D.); ivana.bobeldijk@tno.nl (I.B.-P.); robert.kleemann@tno.nl (R.K.); 2 Human and Animal Physiology, Wageningen University, 6708 WD Wageningen, The Netherlands; 3 Department of Microbiology and Systems Biology, The Netherlands Organization for Applied Scientific Research (TNO), 3704 HE Zeist, The Netherlands; martien.caspers@tno.nl (M.C.); jaap.keijer@wur.nl (J.K.); 4 Center for Proteomics and Metabolomics, Leiden University Medical Center, Albinusdreef 2, 2333 ZA Leiden, The Netherlands; m.heijink@lumc.nl (M.H.); m.a.giera@lumc.nl (M.G.); 5 Aker BioMarine Antarctic AS, Oksenøyveien 10, NO-1366 Lysaker, Norway; andreas.storsve@akerbiomarine.com (A.B.S.); petter-arnt.hals@akerbiomarine.com (P.-A.H.); 6 Department of Vascular Surgery, Leiden University Medical Center, 2333 ZA Leiden, The Netherlands

**Keywords:** krill oil, polyunsaturated fatty acids (PUFAs), obesity, inflammation, oxylipins, adipogenesis

## Abstract

The development of obesity is characterized by the metabolic overload of tissues and subsequent organ inflammation. The health effects of krill oil (KrO) on obesity-associated inflammation remain largely elusive, because long-term treatments with KrO have not been performed to date. Therefore, we examined the putative health effects of 28 weeks of 3% (*w*/*w*) KrO supplementation to an obesogenic diet (HFD) with fat derived mostly from lard. The HFD with KrO was compared to an HFD control group to evaluate the effects on fatty acid composition and associated inflammation in epididymal white adipose tissue (eWAT) and the liver during obesity development. KrO treatment increased the concentrations of EPA and DHA and associated oxylipins, including 18-HEPE, RvE_2_ and 14-HDHA in eWAT and the liver. Simultaneously, KrO decreased arachidonic acid concentrations and arachidonic-acid-derived oxylipins (e.g., HETEs, PGD_2_, PGE_2_, PGF_2_α, TXB_2_). In eWAT, KrO activated regulators of adipogenesis (e.g., PPARγ, CEBPα, KLF15, STAT5A), induced a shift towards smaller adipocytes and increased the total adipocyte numbers indicative for hyperplasia. KrO reduced crown-like structures in eWAT, and suppressed HFD-stimulated inflammatory pathways including TNFα and CCL2/MCP-1 signaling. The observed eWAT changes were accompanied by reduced plasma leptin and increased plasma adiponectin levels over time, and improved insulin resistance (HOMA-IR). In the liver, KrO suppressed inflammatory signaling pathways, including those controlled by IL-1β and M-CSF, without affecting liver histology. Furthermore, KrO deactivated hepatic REL-A/p65-NF-κB signaling, consistent with increased PPARα protein expression and a trend towards an increase in IkBα. In conclusion, long-term KrO treatment increased several anti-inflammatory PUFAs and oxylipins in WAT and the liver. These changes were accompanied by beneficial effects on general metabolism and inflammatory tone at the tissue level. The stimulation of adipogenesis by KrO allows for safe fat storage and may, together with more direct PPAR-mediated anti-inflammatory mechanisms, attenuate inflammation.

## 1. Introduction

Metabolic overload caused by excessive intake of energy-dense foods promotes the development of obesity, which is characterized by inflammation in white adipose tissue (WAT) and the liver [1]. During the development of obesity, WAT expands to store the energy surplus. This WAT expansion involves two processes: adipocyte hyperplasia (an increase in the cell number via adipogenesis) and adipocyte hypertrophy (an increase in cell size) [2]. Both processes have a physiological limit, and these vary between different WAT depots. The epididymal WAT (eWAT) of mice, for example, predominantly responds to metabolic overload with hypertrophy [3]. We and others have shown that the time point of maximal eWAT expansion coincides with the development of adipose tissue inflammation [3,4]. eWAT is thought to be particularly susceptible to becoming inflamed because of its limited ability to initiate the adipogenesis program required for hyperplasia. The development of hypertrophic and inflamed WAT is typically paralleled by changes in systemic inflammation markers, including cytokines (e.g., TNF-α, Il-6) and adipokines [5]. These circulating factors can contribute to the development of insulin resistance as well as liver steatosis and inflammation in obese subjects [6]. Treatments that could correct metabolic imbalances and exert anti-inflammatory activities would be beneficial for all obesity-associated disorders.

Krill oil (KrO), a marine oil extracted from Antarctic krill, is rich in phospholipids (>55% of total lipids), most of which are phosphatidylcholines (>85%). KrO has a high content of long-chain omega-3 polyunsaturated fatty acids (PUFAs), in particular eicosapentaenoic acid (EPA) and docosahexaenoic acid (DHA) [7]. Lipids from KrO can be stored in cells or converted intracellularly into a variety of bioactive oxylipins that can act as direct inflammatory modulators [8]. Many of the fatty acids (FAs) present in KrO, as well as their metabolites, are ligands of transcriptional regulators, including peroxisome-proliferator-activated receptors (PPARs) [9]. PPARα and PPARγ are predominately expressed in the liver and WAT, respectively, where they control the expression of genes that are critical for metabolism and adipogenesis. In addition, PPARs have anti-inflammatory properties, and can repress NF-κB-mediated transcription via multiple mechanisms (including direct physical association and upregulation of the IκBα protein) [10,11,12]. Besides being a ligand for PPARs, KrO-derived EPA and DHA can displace arachidonic acid (ARA), an omega-6 FA with proinflammatory properties, from membranes and may also compete with ARA for further enzymatic processing [13]. Via these indirect mechanisms, EPA and DHA can attenuate the pro-inflammatory effects of ARA and its downstream metabolites, such as hydroxyeicosatetraenoic acids (HETEs), oxoeicosanoids (KETEs), prostaglandins (PGs), thromboxanes (TXs) and leukotrienes (LTs) [14]. Furthermore, EPA and DHA themselves can be enzymatically converted into inflammation-resolving mediators, including hydroxyeicosapentaenoic acids (HEPEs), hydroxy docosahexaenoic acids (HDHAs) and further into resolvins (RVs), protectins (PDs) and maresins (MaRs) [14].

In this study, our aim was to examine the putative health effects of long-term (28 weeks) KrO supplementation (3% *w*/*w*) in mice, since the health effects of KrO on obesity-associated organ inflammation remain largely elusive due to the lack of long-term treatment studies using physiologically relevant KrO concentrations. A small number of relatively short studies (less than 12 weeks of KrO treatment [15,16,17,18,19]) consistently point to an improvement of metabolic homeostasis and metabolic risk factors. To gain a better mechanistic insight into how KrO exerts its effects, we analyzed WAT and the liver by means of genome-wide mRNA sequencing to identify molecular pathways and critical upstream regulators. Furthermore, both tissues were subject to a comprehensive fatty acid composition analysis, including oxylipins using LC-MS/MS. Ldlr−/− Leiden mice were chosen as a model because these mice develop obesity in combination with pronounced hyperinsulinemia and adipose tissue and liver inflammation when treated with energy-dense diets for 28 weeks (to allow for the histological analysis of organ inflammation) with a human-diet-like macronutrient composition, not requiring dietary supplementation with cholesterol [20,21,22,23]. Under the experimental conditions employed herein, Ldlr−/− Leiden mice have been shown to develop histopathological features similar to humans and they recapitulate human disease pathways, as demonstrated by recent comparative transcriptomics, metabolomics and proteomics studies [21,22,24]. We show that long-term KrO treatment markedly affects the tissue levels of PUFAs and oxylipins, and consistently with this, reduces their inflammatory tone.

## 2. Materials and Methods

### 2.1. Animals, Diets and Study Design

All animal experiments were performed in accordance with the Animal Care and Use Committee and ethical approval by an independent Animal Welfare Body (IVD TNO; approval number 3682/TNO-274). Male Ldlr−/− Leiden mice were obtained from the breeding facility of TNO Metabolic Health Research, Leiden, The Netherlands. All mice were group-housed in an AAALAC-accredited animal facility (relative humidity 50–60%, temperature ~21 °C, light cycle 7 am to 7 pm) and had ad libitum access to food and water. Body weight and blood glucose data obtained before the start of dietary treatment were used to match mice into three comparable groups, to ensure that group differences at the end were attributable to the treatment they received during the study and not due to differences at baseline. The control group (*n* = 15) was treated with an energy-dense high-fat diet (HFD; D12451, Research Diets Inc.; 20 kcal% protein, 35 kcal% carbohydrate, 45 kcal% fat, with 39 kcal% from lard and 6 kcal% from soybean oil). The KrO treatment group received an HFD in which 3% of the total diet was replaced with KrO (KrO diet). The KrO (Superba Boost, Aker BioMarine Antarctic ASA, Lysaker, Norway) itself contained EPA ≥150 mg/g and DHA ≥70 mg/g, which is approximately 20% of EPA and DHA fatty acids in KrO. Fatty acids are structural components of lipid species that are efficiently taken up in the intestine (phospholipids). KrO is a marine oil that is particularly rich in phospholipids and the KrO used in this study contained ≥560 mg/g phospholipids, of which ≥480 mg was phosphatidylcholine. At an expected average daily intake of 3 g diet per mouse, the dietary intake of KrO was 0.09 g/mouse/day on average. HFD and KrO diets were isocaloric. A reference group remained on a low-fat control chow diet (*n* = 6) (chow; Ssniff-Spezialdiäten GmbH, Soest, Germany).

Body weight and food intake were measured regularly, and body composition was determined using echoMRI (EchoMRI-LLC, Houston, TX, USA). Blood samples were taken from the tail vein at the start of the study and after 8 weeks, 16 weeks and 28 weeks. In week 28, animals were terminated after 5 h fasting via gradual-fill CO_2_ asphyxiation and a terminal blood sample was collected via cardiac puncture. Isolated adipose and liver tissues were fixed in formalin and paraffin-embedded for histological analysis, or snap-frozen and stored at −80 °C for gene expression, lipid and protein analyses.

### 2.2. Blood Chemistry

Analyses of cholesterol, triglycerides, insulin, leptin, adiponectin, ALAT in fasting EDTA plasma and blood glucose were performed as described previously [22]. The blood sampling over time allowed us to calculate area under the curve (AUC) values of plasma parameters (GraphPad Prism, version 8 for Windows, GraphPad Software, La Jolla, CA, USA, www.graphpad.com, accessed on 23 March 2021). Free fatty acids (FFAs) were measured in orlistat (1 mg/L; Sigma-Aldrich, St. Louis, MO, USA) [25]-treated plasma and quantified with the NEFA-HR kit (Instruchemie, Delfzijl, The Netherlands).

### 2.3. Total Fatty Acid Composition Analysis in Dry Blood Spots, White Adipose Tissue and Liver

Dry blood spots (DBSs) collected during blood sampling, as well as tissue collected at sacrifice from eWAT and the liver, were analyzed for their fatty acid composition (OmegaQuant Analytics, Sioux Falls, SD, USA). Briefly, blood was collected during blood sampling after 28 weeks of treatment on filter paper pre-treated with an antioxidant cocktail (Fatty Acid Preservative Solution) and dried at room temperature for 15 min. Tissue samples were weighed and transferred into screw-cap glass vials that contained tritricosanoin as an internal standard (tri-C23:0 TG) (NuCheck Prep, Elysian, MN, USA). These tissues were homogenized and then extracted with a modified Folch extraction. A portion of the organic layer was transferred to a screw-cap glass vial and dried in a speed vac. The dried tissue samples and a punch of the DBS filter paper in screw-cap glass vials were treated with BTM solution (methanol containing 14% boron trifluoride, toluene, methanol; 35:30:35 *v*/*v*/*v*) (Sigma-Aldrich). The vial was briefly vortexed and heated (100 °C for 45 min). After cooling, hexane (EMD Chemicals, New Hampshire, MA, USA) and HPLC-grade water were added, and samples were vortexed and centrifuged to separate layers. An aliquot of the hexane layer was used for gas chromatography (GC). GC was carried out using a GC-2010 (Shimadzu Corporation, Columbia, MD, USA) equipped with a SP-2560, 100-m fused silica capillary column (0.25 mm internal diameter, 0.2 µm film thickness; Supelco, Bellefonte, PA, USA). Fatty acids were identified and calibrated using a standard mixture of defined fatty acids (GLC OQ-A, NuCheck Prep). Fatty acid composition was expressed relatively (percentage of total identified fatty acids) and given in absolute concentrations (µg fatty acid per mg WAT or liver tissue).

### 2.4. Free Fatty Acids and Lipid Mediator Composition in White Adipose Tissue and Liver

White adipose and liver tissues from sacrificed mice were homogenized in water at a concentration of 0.33 mg/µL (Biosolve, Valkenswaard, The Netherlands). WAT (10 mg) or liver tissue (2.7 mg) homogenates were mixed with 600 µL MeOH (Merck, Darmstadt, Germany) containing internal standards (0.33 ng/mL PGE_2_-d4, LTB_4_-d4, 15-HETE-d8, 14(15)-EET-d11, 0.66 ng/mL 8-iso-PGF_2_ alpha-d4 and 3.3 ng/mL DHA-d5, all Cayman Chemical, Ann Abor, MI, USA) and 200 µL water was added to the mixture. Samples were incubated for 20 min at −20 °C and centrifuged (10 min at 16.100× *g*, 4 °C). Supernatant was diluted with water, acidified to pH ±3.0 using formic acid (VWR, Darmstadt, Germany) and applied to C18 SPE cartridges (Sep-Pak C18, 200 mg, 3 cc, Waters, Milford, MA, USA). The obtained samples were cleaned consecutively with water and *n*-hexane (VWR) and eluted using methyl formate (Sigma-Aldrich). The eluate was dried at 40 °C under a stream of nitrogen and reconstituted in 40% MeOH. Samples were analyzed using an LC-MS/MS system consisting of two LC-30AD pumps, a SIL-30AC autosampler and a CTO-20AC column oven (All Shimadzu, Hertogenbosch, The Netherlands). Samples were injected with an autosampler (at 6 °C) and separated on a Kinetex C18 column (Phenomenex, Aschaffenburg, Germany, 50 × 2.1 mm, 1.7 µm) using a gradient of 0.01% acetic acid (Fluka, Darmstadt, Germany) in water (Honeywell-Riedel de Haën, Seelze, Germany; eluent A) and 0.01% acetic acid in MeOH (eluent B) with the oven at 50 °C. The gradient was as follows: 0.0–1.0 min constant at 30% B, 1.0–1.1 min linear increase to 45% B, 1.1–2.0 min linear increase to 53.5% B, 2.0–4.4 min linear increase to 55.5% B, 4.0–7.0 min linear increase to 90% B, 7.0–7.1 min linear increase to 100% B, 7.1–9.0 min constant at 100% B, 9.0–9.5 min linear decrease to 30% B, 9.5–11.5 min constant at 30% B. Detection was achieved on a Qtrap 6500 (Sciex Nieuwerkerk a/d, IJssel, The Netherlands) equipped with an ESI source. The MS was operated in negative scheduled MRM mode, with the source needle voltage at −4500 V, drying temperature of 450 °C, ion source gas 1 and 2 (both air) at respectively 40 and 30 psi and the nitrogen nebulizer gas at 30 psi. The entrance potential was set to 10 V and the collision gas flow to ‘medium’. Individually optimized parameters for each compound can be found in Supplementary Table S1.

The detected lipid mediators PDX and RvE_2_ were identified based on comparisons with authentic standard material. Both substances showed closely matching (<0.5% deviation) relative retention times between samples and authentic standard material. Furthermore, the tandem mass spectra for PDX were closely matched (Supplementary Figure S1). In the case of RvE_2_, we monitored four characteristic fragment ions of RvE_2_ by means of a dedicated product ion scan LC-MS/MS method. The obtained relative retention times and ion ratios were also compared with authentic standard material (Supplementary Figure S2). Standards of RvE_1_, RvE_2_, 18(R)-RvE_3_ and 18(S)-RvE_3_ were gifts from Dr. Makoto Arita (Tokyo, Japan); all other standards were obtained from Cayman Chemicals.

### 2.5. Histological Analysis of Adipose Tissue and Liver

Paraffin-embedded 5-µm-thick cross sections of eWAT were stained with hematoxylin-phloxine-saffron and scanned for digital analysis (Aperio AT2, Leica Biosystems, Amsterdam, The Netherlands). Cell size and counts of adipocytes were analyzed using Adiposoft [26]. Inflammation was quantified by scoring the amount of crown-like structures (CLS) and expressed as the number of CLS/1000 adipocytes, as described previously in detail [27]. The calculation of total eWAT cell numbers as a measure of hyperplasia was performed using a well-established mathematical model [28].

The histopathological analysis of liver steatosis and hypertrophy was performed on 3-µm-thick hematoxylin-eosin-stained cross sections of the medial lobe using a standardized method for rodent liver histopathology, based on the human NAS scoring system [29,30]. The percentages of the total liver sections affected by steatosis and hypertrophy translate into the NAS categories of severity as follows: 0 (<5%), 1 (5–33%), 2 (34–66%) and 3 (>66%) [29]. In addition, F4/80 immunostaining was performed on the liver sections as detailed in [21] and the number of F4/80-positive CLSs was counted in three nonoverlapping fields and expressed per mm^2^.

### 2.6. Liver Biochemistry

Total hepatic triglyceride content was determined in crude liver homogenates by following the Bligh and Dyer method for lipid extraction with methanol and chloroform [31]. In the same liver homogenates, protein levels were determined, using a Lowry protein assay. The lipids were separated by means of high-performance liquid chromatography (HPTLC) on silica gel plates and stained with color reagent (5 g of MnCl_2_•4 H_2_O, 32 mL of 95–97% H_2_SO_4_ added to 960 mL of CH_3_OH:H_2_O 1:1 *v*/*v*). The liver triglyceride bands were quantified with a ChemiDoc Touch Imaging System (Bio-Rad, Hercules, CA, USA) using Image-lab version 5.2.1 software (Bio-Rad) and were expressed per mg protein.

### 2.7. Genome-Wide Gene Expression Analysis

Next generation sequencing (NGS) was performed essentially as described [21]. Briefly, total RNA was extracted from eWAT using an Ambion RNAqueous total RNA isolation kit (Thermo Fisher Scientific, Waltman, MA, USA) and from the liver using the RNA-Bee total-RNA isolation kit (Bio-Connect, Huissen, The Netherlands), and was subsequently purified using PureLink RNA Mini Kit (Thermo Fisher Scientific). The RNA concentration was determined spectrophotometrically using a Nanodrop 1000 (Isogen Life Science, De Meern, The Netherlands), and RNA quality was assessed using a 2100 Bioanalyzer (Agilent Technologies, Amstelveen, The Netherlands). Strand-specific messenger RNA sequencing libraries were multiplexed, clustered and sequenced on a Nextseq500 V2 system (Illumina, San Diego, CA, USA) at Genomescan (Leiden, The Netherlands). The sequenced, annotated and aligned gene counts served as inputs for the differentially expressed gene (DEG) analysis using the Deseq2-method [32]. The integrity of the gene expression datasets from eWAT and the liver were analyzed (e.g., distance matrix and principal component analysis) and 10% technical outliers were excluded. For the subsequent bioinformatical analysis, *n* = 5 chow, *n* = 9 HFD and *n* = 9 KrO liver samples, as well as *n* = 5 chow, *n* = 8 HFD and *n* = 10 KrO eWAT samples, were used. DEGs were used as inputs for pathway analysis through Ingenuity Pathway Analysis (IPA; www.ingenuity.com, accessed on 23 March 2021). IPA uses gene expression data of all known downstream target genes to predict the activation or deactivation of an upstream regulator. Z-scores greater than 2 indicate the enhanced activity of an upstream regulator, whereas a Z-score less than −2 indicates the reduced activity of an upstream regulator [21]. The *p*-value indicates the significance of the gene enrichment of these upstream regulators.

### 2.8. Western Blot Analysis

Liver tissue from HFD- and KrO-treated mice (*n* = 4/group) were homogenized in lysis buffer containing 150 mM NaCL, 1 mM EDTA PH = 8, 50 mM Tris-HCL PH = 7.4, 1% Igepal, 0.25% deoxycholate, 0.1% SDS, 1 mM PMSF, 1 mM Na_3_VO_4_ and complete mini EDTA-free protease inhibitor (Roche, Mannheim, Germany). Homogenates were centrifuged at 13,000 rpm for 15 min at 4 °C, and the protein content of the supernatant was determined using the BCA Protein Assay Kit (Thermo Fisher Scientific). Proteins (50 ug) in 2× SDS Laemmli Sample Buffer (1:1 *v*/*v*; Sigma-Aldrich) were boiled for 5 min at 95 °C. Subsequently, samples were separated on a 4–20% (*w*/*v*) SDS-page gel (mini-Protean TGX stain-free precast gels; Bio-Rad). Proteins were transferred onto Trans-blot Turbo mini PVDF blotting membranes (Bio-Rad) using the MIXED MW program on the Trans-blot Turbo Bio-Rad machine. The blotting membranes were blocked for 1 h with 5% (*w*/*v*) milk powder in tris-buffered saline with 0.1% Tween 20 and incubated overnight at 4 °C with either the primary antibody IκBα (#9242S–1:1000 *v*/*v*; Cell Signaling, Leiden, The Netherlands), PPARα (ab24509–1:1000 *v*/*v*; Abcam, Cambridge, UK) or αTubulin (T5168-1:1000 *v*/*v*; Sigma-Aldrich). Secondary antibody (anti-mouse HRP conjugate #7076S-1:2000 *v/v* or anti-rabbit HRP conjugate #7074S-1:2000 *v*/*v*; Cell Signaling) was added and SuperSignal West Femto (Thermo Fisher Scientific) was used to visualize protein bands. Blots were analyzed with a ChemiDoc Touch Imaging system (Bio-Rad) and band intensities were normalized to αTubulin.

### 2.9. Statistical Analysis

All data shown are presented as mean ± standard deviation (SD). The experiments investigated the null hypothesis that KrO does not have beneficial effects on disease parameters relative to controls (HFD). Therefore, KrO was compared to the HFD control group, and data from chow mice were provided as a reference. The significance of differences between KrO and HFD was tested using one-sided *t*-tests (α = 0.05) if the data were normally distributed with equal variances. Data sets that were not normally distributed or with unequal variances were tested with a Mann–Whitney U test. IPA analysis to determine differentially expressed genes were based on Fisher’s exact test (α = 0.01).

## 3. Results

### 3.1. Long-Term KrO Treatment Improves Lipid Composition in Blood, eWAT and Liver Tissue

During 28 weeks of HFD feeding, body weight increased from 29.4 g to 52.7 g in the HFD control group. EchoMRI analysis showed that the observed weight gain relative to chow could mainly be ascribed to an increase in fat mass (Table 1). KrO treatment had no effect on body weight development, total fat mass or absolute mass of eWAT or subcutaneous WAT (sWAT), whereas the mesenteric WAT (mWAT) mass was significantly lower than in HFD. The energy intake was comparable in all groups. Plasma lipids were significantly elevated by the HFD compared with chow, indicating a state of hyperlipidemia, and were not affected by KrO (Table 1).

To assess whether KrO altered the omega-3 FA content in the circulation, we analyzed the FA composition in dry blood spots (Supplementary Table S2). Compared to HFD, KrO treatment significantly increased the relative amounts of omega-3 FAs, with marked effects on EPA (19.8-fold) and DHA (1.5-fold).

We next examined whether similar changes would occur at the tissue level (Table 2). In eWAT, KrO treatment increased the absolute tissue concentrations of EPA (13.7-fold) and DHA (3.6-fold) and other omega-3 FAs measured. KrO had comparable effects in the liver, where it increased EPA (10.8-fold) and DHA (2.0-fold) concentrations. Table 2 also demonstrates that the absolute concentrations of EPA and DHA were lower in eWAT than in the liver.

By contrast, KrO treatment significantly reduced ARA concentrations in the circulation by 59% compared to HFD (0.4-fold reduction), i.e., below the levels of chow reference mice (Supplementary Table S2). In WAT and the liver, KrO had similar effects and strongly reduced ARA tissue concentrations by 59% and 52%, respectively. Other omega-6 FAs were also lowered by KrO, with the exception of linoleic acid and gamma-linolenic acid. The concentrations of monosaturated, saturated and trans FAs were scarcely changed by KrO.

Collectively, these data indicate that KrO treatment affected the lipid composition in the circulation, eWAT and the liver in a comparable way: all tissues showed a marked increase in omega-3 FAs and a decrease in ARA concentrations. The highest absolute concentrations of omega-3 FAs were observed in the liver.

### 3.2. KrO Reduces WAT Inflammation, Improves Adipocyte Size Distribution and Increases Inflammation-Resolving Lipid Mediators Derived from EPA and DHA

We next investigated the possible effects of KrO on WAT histology, and analyzed adipocyte cell size, WAT inflammation (CLS) and the total number of adipocytes in eWAT.

**Figure 1:**
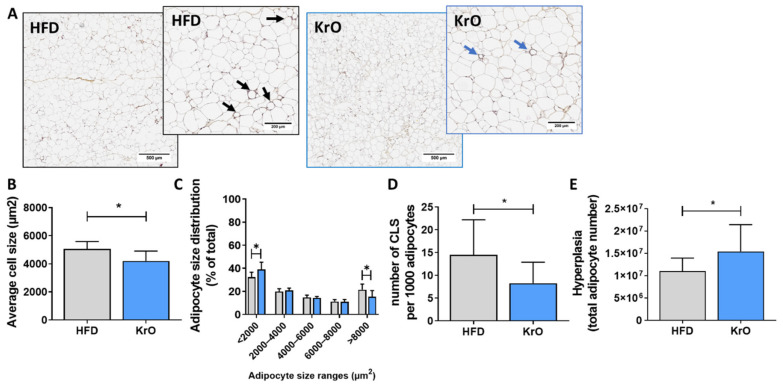
Effect of KrO on eWAT. (**A**) Representative images of HPS-stained eWAT, one low magnification image and one high magnification image per treatment, with arrows to indicate crown-like structures (CLS). eWAT sections were analyzed to determine (**B**) average adipocyte cell size, (**C**) adipocyte size distribution, (**D**) number of CLS per 1000 adipocytes, and (**E**) total number of eWAT cells as a measure of hyperplasia. Data are presented as mean ± SD, * *p* < 0.05 compared to HFD.

The average adipocyte cell size of the HFD control group (5063.1 ± 518.7 µm^2^) was similar to that of the chow reference group (4724.4 ± 891.1 µm^2^). Treatment with KrO significantly reduced the average adipocyte size (4200.1 ± 709.6 µm^2^) relative to HFD (Figure 1A,B). A subsequent, more refined, analysis of the cell size distribution revealed that this effect was attributable to an increase in the number of very small adipocytes (<2000 µm^2^) by KrO, whereas the number of large adipocytes (>8000 µm^2^), which reportedly release many inflammatory mediators [33], decreased (Figure 1C).

In the HFD control group, many immune cells were observed, and a substantial portion of inflammatory cells formed CLSs (14.5 ± 7.7 per 1000 adipocytes). The number of CLSs in HFD was strongly increased relative to chow (2.2 ± 4.8 CLSs per 1000 adipocytes). Treatment with KrO prevented HFD-induced CLS formation and significantly reduced CLS numbers by approximately 42% (Figure 1D), demonstrating that KrO reduces tissue inflammation. This histological effect was substantiated by the results of a genome-wide gene expression analysis and subsequent upstream regulator analysis of eWAT. This analysis predicts the activation state of an upstream regulator (e.g., cytokines, signaling molecules, transcription factors) based on the expression pattern of genes downstream from this regulator. HFD activated multiple upstream regulators relative to chow, including TNFα, TRAP1, CCL2/MCP-1, IL-1β, IL-6 and MIF, indicating the activation of a broad spectrum of proinflammatory signaling pathways. KrO significantly suppressed many of these inflammatory pathways, as demonstrated by the deactivation of the respective upstream regulators/cytokines (Table 3).

**Figure 2:**
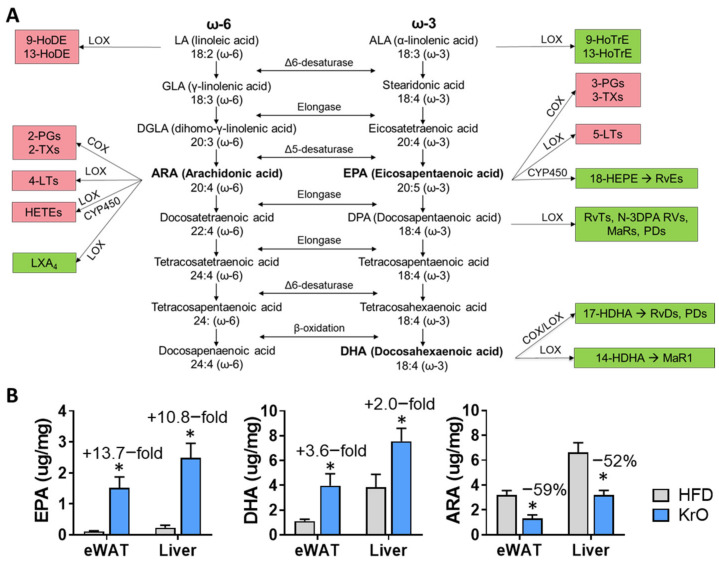
(**A**) Overview of omega-6 (ω-6) and omega-3 (ω-3) FA synthesis pathways with linoleic acid (LA) and α-linolenic acid (ALA) constituting the upstream precursors, respectively. PUFAs compete for the same desaturase and elongase enzymes during the synthesis of eicosapentaenoic acid (EPA), docosahexaenoic acid (DHA) and arachidonic acid (ARA). These PUFAs can further be metabolized by the COX/LOX/CYP450 enzymes into oxylipins. Oxylipins that are typically implicated in the potentiation of inflammatory responses are highlighted in red, whereas oxylipins typically involved in the resolution of inflammatory responses are shown in green. (**B**) Effect of KrO on the tissue concentrations of EPA, DHA and ARA, determined by GC. Data are presented as mean ± SD, * *p* < 0.05 compared to HFD.

Intracellular free PUFAs and their respective oxylipin metabolites directly involved in regulating inflammatory responses were measured in eWAT homogenates. A schematic overview of oxylipins that can be synthesized from EPA, DHA and ARA is provided in Figure 2A, together with the tissue concentrations of these three precursors (Figure 2B). Feeding with the HFD lowered the levels of omega-3-derived mediators 13-HoTre and 19,20 DiHDPA and increased 18-HEPE and 4-HDHA, 10-HDHA and 17-HDHA (Table 4). Simultaneously, HFD increased intracellular ARA and many pro-inflammatory omega-6-derived oxylipins, including 9-HoDE, 8–11- and 15-HETE and prostaglandin D2 (PGD_2_). By contrast, KrO upregulated all EPA- and DHA-derived oxylipins, whereas almost all omega-6 derived pro-inflammatory oxylipins were significantly suppressed with the KrO diet (Table 4).

Pathway analysis of the gene expression data also revealed that apelin signaling, an insulin-dependent adipocyte differentiation pathway [34], was strongly activated by KrO and that the activity of important upstream regulators of adipogenesis, including PPARγ, RB1, CEBPα, KL4, KL15, STAT5A and STAT5B (Table 3), was stimulated by KrO. Conversely, negative regulators of differentiation, such as KDM5A and STK11, were activated by HFD and suppressed with KrO. To corroborate this finding with histology results, we calculated the total number of adipocytes present in eWAT using an established method [28]. The adipocyte number in eWAT of HFD-fed animals (1.10 × 10^7^) was similar to that of the chow reference group (1.08 × 10^7^). Interestingly, KrO-fed mice showed a significant 1.4-fold increase in adipocyte numbers (1.54 × 10^7^), which is clearly indicative of hyperplasia (Figure 1E) and which supports the results of the upstream regulator analysis.

Furthermore, HFD deactivated PPARGC1A, PPARGC1B, ESRRA and NRF1, whereas KrO strongly induced these upstream regulators, pointing to a beneficial effect on mitochondrial biogenesis.

Collectively, these data show that treatment with KrO reduced CLSs and increased the number of adipocytes in eWAT. In line with these histological results, KrO prevented the activation of inflammatory regulators by HFD, increased PUFA concentrations and stimulated the formation of anti-inflammatory bioactive lipids. KrO treatment also resulted in a shift towards smaller adipocytes and fewer large adipocytes, possibly as a consequence of its adipogenesis-stimulating effect.

### 3.3. Effect of KrO on Adipokines and Metabolic Risk Factors

We next investigated whether the observed histological changes in WAT were accompanied by effects on adipokine levels and insulin resistance (HOMA-IR).

Plasma leptin levels were 2.5 ± 2.0 ng/mL at t = 0 and were increased by HFD, reaching 58.1 ± 10.8 ng/mL in week 28, which differed significantly from the chow reference group (26.6 ± 8.0 ng/mL). KrO significantly attenuated this HFD-evoked increase in leptin (37.7 ± 6.4 ng/mL). Furthermore, the total leptin exposure over time (AUC from data at 0, 8, 16 and 28 weeks) was significantly reduced by KrO (Table 5). Plasma adiponectin levels were higher with KrO compared to HFD at the beginning of the treatment until week 16 and this difference became smaller towards the end of the study. The total adiponectin exposure over time (AUC) was significantly higher in KrO compared to HFD (Table 5).

Fasting blood glucose levels and HFD-induced increases in plasma insulin levels were reduced with KrO. Subsequently, the HOMA-IR values were significantly lower in the KrO group, indicating that KrO improved insulin resistance. Genome-wide gene expression analysis showed that HFD inactivated upstream regulators that are important in insulin signaling (INSR and IGF1R) in both the liver and eWAT, and KrO counteracted this effect in eWAT (Table 3).

Free fatty acid (FFA) plasma concentrations were not significantly raised with HFD, whereas KrO significantly increased FFAs compared to the HFD in line with the lower insulin concentrations.

Plasma ALT and AST levels were significantly increased by the HFD compared to the chow reference diet. This increase in both ALT and AST tended to be less pronounced in the KrO group.

Taken together, KrO reduced leptins and increased adiponectin exposure, which was paralleled by an improvement in HOMA-IR.

### 3.4. Effects of Krill Oil in the Liver

**Figure 3:**
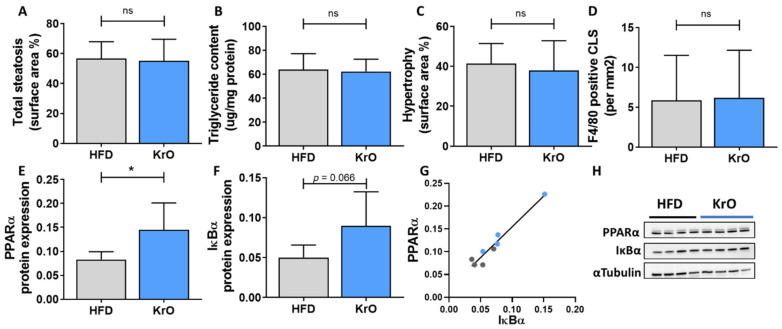
Effect of KrO on the liver—(**A**) total steatosis, (**B**) triglyceride content expressed per mg of liver protein (**C**) hepatocellular hypertrophy and (**D**) F4/80-positive crown-like structures (CLSs). Liver protein levels of (**E**) PPARα and (**F**) IκBα normalized for αTubulin expression, and (**G**) correlation between PPARα and IκBα. (**H**) representative images of the Western blot bands. Data are presented as mean ± SD, * *p* < 0.05 compared to HFD.

HFD treatment resulted in the development of hepatic steatosis, hepatocellular hypertrophy and the formation of F4/80-positive CLSs, indicating macrophage aggregation around dying hepatocytes (Figure 3A–D). These histological characteristics were practically absent in the chow group. The liver histology and the lipid content of the KrO group was comparable to that of the HFD group. On a molecular level, however, gene expression profiling and subsequent upstream regulator analysis demonstrated the activation of many proinflammatory pathways by HFD, which were reversed by KrO (Table 6). More specifically, KrO quenched the HFD-induced activation of proinflammatory cytokine signaling (e.g., by IL-1β, IFN-A2 and CSF1) and proinflammatory transcription factors (e.g., REL-A/p65-NF-κB and STAT1). Critical upstream regulators of metabolism, including the β-oxidation enzyme ACOX1 and the mitochondrial biogenesis activator PPARGC1A, were deactivated with HFD, and KrO attenuated this effect, suggesting a reactivation of lipid processing. Moreover, HFD activated SREBF1 as key regulator of de novo lipogenesis, whereas KrO deactivated SREBF1 and thereby seemed to suppress lipid synthesis. Furthermore, cholesterol synthesis regulators deactivated by HFD (e.g., SCAP, SREBP2) were even further suppressed with KrO (Table 6).

When compared to its anti-inflammatory activities in WAT, KrO attenuated different pro-inflammatory pathways and a different set of transcriptional regulators in the liver. Many of the KrO-modulated signaling pathways in the liver converge in transcriptional regulation by NF-κB family members, and KrO significantly deactivated REL-A/p65-NF-κB, for instance. In line with this, Western blot analysis of liver tissue homogenates revealed an increase in PPARα protein expression and a trend (*p* = 0.066) towards an increase in IκBα protein levals by KrO relative to HFD. PPARα protein expression was significantly correlated with IκBα protein expression (R^2^ = 0.9) (Figure 3D–F).

Next, we investigated whether KrO affected the intrahepatic levels of bioactive lipids using the same oxylipin platform as before. Table 7 shows that HFD significantly decreased the levels of ALA-, EPA- and DHA-derived lipid mediators (e.g., 15- and 18-HEPE, involved in inflammation resolution), while increasing many LA- and ARA-derived lipid species known to promote inflammation (e.g., HETEs, PGD_2_, PGE_2_, PGF_2_ and TXB_2_). By contrast, KrO-treated livers exhibited lower levels of ALA and higher levels of LA, suggesting that the processing of omega-3 FAs is enhanced in the presence of KrO. Indeed, all detectable EPA- and DHA-derived metabolites (except 14-HDHA, a precursor of MaR_1_) were significantly increased with KrO and a strong signal corresponding to genuine resolvin RvE_2_ (same MS/MS transition, identical retention time) was only detectable in KrO-fed mice. Strikingly, KrO suppressed the formation of oxylipins with proinflammatory properties downstream of ARA to such an extent that they were no longer detectable.

Altogether, KrO quenched inflammatory signaling pathways and upregulated the expression of proteins that can interfere with NF-κB signaling (e.g., PPARα). It also suppressed the accumulation of lipids and oxylipins with pro-inflammatory properties and strongly induced the presence of lipid species that contribute to an anti-inflammatory milieu.

## 4. Discussion

This study shows that the fatty acid and oxylipin content of adipose tissue and the liver can be strongly modulated with KrO during the development of obesity, and that KrO treatment attenuates inflammatory processes, resulting in a reduced inflammatory tone. Lipidomic analyses, in conjunction with functional regulator analyses of signaling pathways in adipose tissue and the liver, provided rationales for possible direct and indirect mechanisms by which KrO exerts its effects. One of the direct effects is the increase in anti-inflammatory oxylipins. An important indirect effect is the activation of adipogenic gene expression programs in adipose tissue, accompanied by WAT hyperplasia. The observed increase in WAT storage capacity and the associated decrease in inflammation can contribute to whole-body metabolic health, which is consistent with the observed improvement in insulin resistance (i.e., HOMA-IR) and adipokine concentrations.

A general FA compositional analysis revealed that KrO strongly increased the omega-3 PUFA content in the circulation, WAT and the liver (especially that of EPA and DHA), while simultaneously decreasing the omega-6 PUFA content in these organs, with pronounced reductions in ARA. The observed fatty acid composition changes were grosso modo comparable for both organs, yet the absolute omega-3 PUFA concentrations per milligram of tissue were clearly higher in the liver. Importantly, a refined oxylipin LC-MS/MS analysis showed that KrO strongly increased lipid species with anti-inflammatory properties [35,36,37] derived from EPA and DHA (e.g., 18-HEPE, RvE_2_, 14-HDHA, 17-HDHA, PDX/10S,17S-diHDHA), whereas it suppressed the HFD-induced formation of ARA-derived oxylipins (e.g., HETEs, PGD_2_, PGE_2_, PGF_2_ and TXB_2_), which often exert pro-inflammatory effects [37,38,39,40,41]. Notably, the results of the two independent lipidomics approaches employed herein (the fatty acid composition analysis and the oxylipin analyses) were in line with each other. Both approaches consistently indicated that KrO treatment altered the organ lipid environment in favor of PUFAs and oxylipins that can resolve inflammation, while lowering ARA and ARA-derived oxylipins that can promote inflammation. For instance, HFD-feeding increased PGD_2_, PGE_2_ and PGF_2_ levels in both WAT and the liver, which can stimulate chemotaxis of immune cells [42,43] by increasing local cytokine release and the endothelial expression of adhesion molecules to facilitate immune cell tissue infiltration [38,44]. KrO blunted this HFD-evoked effect, and the observed PGD_2_, PGE_2_ and PGF_2_ levels were even lower with KrO compared to the chow diet. Conversely, HFD feeding diminished the levels of anti-inflammatory oxylipins such as 18-HEPE, a precursor for E series resolvins, and 17-HDHA in liver [36]. KrO counteracted this detrimental effect, resulting in an increase of 18-HEPE and 17-HDHA levels in WAT and the liver. Notably, 17-HDHA treatment has been shown to reduce WAT inflammation, increase adiponectin expression and improve glucose tolerance in obese mice [36]. 18-HEPE, RvE_2_, 14-HDHA, 17-HDHA, PDX and other anti-inflammatory oxylipins were increased with KrO in this study and are thought to be directly involved in inflammation resolution mechanisms, as reported by [35,36,37,45,46,47,48], and hence may contribute to a lipid environment in WAT and the liver that protects against chronic inflammation.

It is likely that inflammation-resolving oxylipins were formed from their precursor fatty acids present in KrO (EPA and DHA), but these precursors themselves can also be enzymatically synthesized from ALA. The enzymes responsible for omega-3 PUFA processing are the same as those for omega-6 processing but the rate-limiting enzyme, delta-6-desaturase, has a higher preference for omega-3 FAs relative to omega-6 FAs [49,50]. Thus, an enhanced conversion of ALA in the presence of KrO may also explain the pronounced increases in anti-inflammatory oxylipins.

The observation that the levels of most PUFAs and oxylipins are higher in the liver than in WAT is consistent with metabolic studies in mice [51]. These tissue differences may have multiple causes, including the expression or activity of the aforementioned enzymes that ultimately determine the extent to which substrates (EPA, DHA, ARA) are available for subsequent enzymatic conversions (e.g., by Cyp450, COX, LOX and auto-oxidation) into the respective oxylipins [41,52]. Collectively, the lipidomic analyses demonstrate that KrO can directly affect the inflammatory milieu in WAT and the liver by modulating PUFA and oxylipins in these organs, suggesting beneficial effects on inflammatory pathways and processes.

Indeed, pathway and upstream regulator analyses clearly demonstrated a suppression of HFD-induced inflammatory pathways by KrO in WAT and the liver. For example, KrO deactivated multiple inflammatory HFD-stimulated pathways, including those controlled by TNFα, TNF receptor-associated protein 1 (TRAP1) and CCL2 in eWAT, as well as the IL-1β and M-CSF pathways in the liver. Attenuation of TNFα/IL-1β/CCL2-mediated inflammation in metabolic organs has been shown to improve insulin resistance [53,54,55], consistent with the observed reduction of fasting insulin and HOMA-IR in KrO-treated mice. For instance, TNFα inhibits insulin signaling by stimulating the phosphorylation of inhibitory residues on the insulin receptor substrates (IRS1 and IRS2) and impairs IRS-mediated phosphatidylinositol 3-kinase activation [56,57]. KrO appeared to interfere with this process in our study, because we observed significantly increased upstream regulator activities of INSR, IGF1R and IRS1 in eWAT. This effect is consistent with reduced CLS counts in eWAT and was paralleled by decreased leptin and increased adiponectin concentrations in plasma. The KrO-dependent adipokine changes point to an improvement in whole-body homeostasis, including the neuroendocrine circuits affecting adipocytes [58]. Observed elevations in adiponectin can directly affect immune cells, mediating anti-inflammatory activities [59], as well as affecting general metabolism by inhibiting gluconeogenesis and stimulating lipid utilization/b-oxidation [60,61], which is consistent with our pathway analyses.

The deactivation of TRAP1 by KrO points to reduced oxidative-inflammatory stress in eWAT because TRAP1 is a mitochondrial heat shock protein involved in the maintenance of mitochondrial integrity under stress or pathological conditions [62].

In addition, KrO stimulated the activity of PPARγ, RB1, CEBPα, KLF15 and STAT5A, all of which are transcriptional master regulators that orchestrate adipogenesis [63,64], and it suppressed negative regulators of adipogenesis (KDM5A and STK11). Consistently with the KrO-induced proadipogenic gene expression program in eWAT, the absolute number of adipocytes increased in this depot, and so did the proportion of small adipocytes, together indicating KrO-induced hyperplasia. WAT hyperplasia constitutes a mechanism by which the total storage capacity for fat is increased, allowing the body to cope with metabolic overload from HFD [3]. This indirectly prevents adipocyte hypertrophy and tissue inflammation and attenuates the effect of cytokines implicated in insulin resistance (e.g., TNFα and MIF signaling [4]), as observed herein. Furthermore, KrO also activated PGC1A, PGC1B, ESSRA and NRF1, which are indicative of increased mitochondrial biogenesis (growth and division of pre-existing mitochondria) in eWAT, which is in line with the reported effects of PUFAs during adipocyte differentiation [65,66]. The activation of PPARγ by KrO-derived lipids may, at least partly, explain the effects observed in eWAT, as well as the observed increase in adiponectin, a PPARγ target gene [59,67]. PPARγ activation could also be responsible for the decrease in leptin, because PPARγ antagonizes CEBPα-mediated leptin mRNA transcription [68]. The activation of PPARγ and PPARα by PUFAs and oxylipins from KrO may contribute to the anti-inflammatory effects observed in WAT and the liver. Activated PPARs can negatively interfere with proinflammatory transcription factors such as NF-κB in several ways: PPARs can directly interact with NF-κB proteins [69] or increase the cytosolic inhibitor, IκBα, thereby preventing nuclear translocation and NF-κB-mediated gene expression [10,11]. The observed increase in PPARα protein levels and high concentrations of potential ligands in KrO-treated livers support the possibility of anti-inflammatory mechanisms involving direct PPARα interaction, [69] and complementary indirect mechanisms (e.g., an increase in IκBα) that negatively interferes with NF-κB signaling. Although KrO attenuated CLS formation in eWAT, there was no effect on F4/80-positive CLSs in the liver. This may be explained by a fundamental difference in plasticity between these organs: although the WAT can respond with hyperplasia and expand further (or redistribute fat among its depots) [70], the liver is anatomically restricted, with a limited storage capacity so that even lipids that are beneficial in nature may cause physical damage to cells when present in excess amounts. In addition, the higher circulating FFAs with KrO may result in a higher influx of fat to the liver, thereby counteracting the beneficial effects on hepatic fat oxidation (i.e., PPARA, ACOX1, PPARGC1A), altogether leading to comparable hepatic steatosis as in the HFD controls. Of note, the steatosis observed in the KrO group is unlikely to be a consequence of increased de novo lipogenesis, because KrO significantly suppressed SREBF1 activity—the key regulator of hepatic lipid synthesis. This is also in line with the observed reduced fasting insulin concentrations with KrO. By contrast, at least a part of the steatosis observed in the HFD control group may be due to increased *de novo* lipogenesis because SREBF1 is significantly activated, and insulin, the transcriptional inducer of Srebp1c, is elevated.

This study has limitations and strengths. Intrinsic to the set-up of the study, we cannot distinguish whether the replacement of HFD by KrO *per se*, or specific KrO components or metabolites formed from KrO, or a combination of the above, are responsible for the beneficial effects observed in KrO-treated mice. We could have included additional groups to substantiate these findings, for instance, groups in which KrO was administered orally superimposed on the HFD. Another limitation is that we cannot estimate the contribution of the PUFAs attached to phospholipids relative to those in other lipid classes. It is possible that the profound effects of KrO are related to its high phospholipid content and the increased bioavailability of EPA and DHA [71], which consequently also affects oxylipin availability. Important strengths of this study are the long period of KrO treatment (28 weeks), resulting in relevant endpoints; the use of large treatment groups (*n* = 15 mice) for high statistical power; the extensive molecular (omics) analyses of adipose tissue and the liver using next-generation sequencing technology to identify activated pathways and upstream regulators in both organs; the profiling of fatty acids in three compartments (plasma, adipose tissue, liver), which provides information on the relative enrichment of PUFAs in each of these tissues; and one of the most comprehensive oxylipin analysis reported so far, including adipose tissue and the liver. Our oxylipin profiling analyses at the tissue level showed that HFD treatment markedly increased oxylipins with proinflammatory properties, whereas oxylipins with inflammation-resolving properties were reduced. KrO treatment appeared to counteract these HFD-evoked changes. These KrO-dependent changes of the inflammatory tone within eWAT and the liver can, at least partly, be explained by the ingested PUFAs, and similar effects may occur in other species. In a human study, it has been shown that a linear dose–response relationship exists between the intake of EPA and DHA and the levels of anti-inflammatory oxylipins measured in plasma [72]. This suggests that the direct effects of KrO on oxylipin tissue concentrations observed herein may also be achievable with dietary regimens in humans.

## 5. Conclusions

We have demonstrated that long-term KrO treatment (28 w) improves the fatty acid composition in the circulation, WAT and the liver. This improvement is characterized by marked elevations in omega-3 FAs and associated oxylipins, which can exert beneficial effects on metabolism (e.g., fasting insulin, adipokines) and tissue inflammation. Functional gene expression and pathway analyses, combined with lipidomic analyses, provide rationales for direct and indirect effects of KrO-derived PUFAs and oxylipins on metabolic and inflammatory pathways controlled by PPARα/PPARγ. The stimulatory effect of KrO on adipogenesis and hyperplasia allows for the safe storage of excess fat in adipose tissue and contributes indirectly to the suppression of inflammation. This indirect effect complements the more direct mechanisms that intercept inflammatory pathways on the molecular level.

## Figures and Tables

**Table 1:** Body composition, energy intake and plasma lipids.

	Chow	HFD	KrO
BW (g)	41.6 ± 6.6 *	52.7 ± 4.4	50.9 ± 2.9
Fat mass (g)	11.0 ± 3.3 *	23.2 ± 2.9	21.4 ± 2.4
Lean mass (g)	29.0 ± 3.6 *	29.6 ± 2.7	29.9 ± 1.8
eWAT (g)	1.9 ± 0.5	2.0 ± 0.5	1.9 ± 0.3
sWAT (g)	1.1 ± 0.3 *	2.5 ± 0.6	2.4 ± 0.4
mWAT (g)	0.7 ± 0.3 *	1.2 ± 0.3	0.9 ± 0.2*
EI (kcal/mouse/day)	13.1 ± 1.1	13.9 ± 1.1	14.2 ± 0.5
FI (g/mouse/day)	4.3 ± 0.4 *	2.9 ± 0.2	3.0 ± 0.1
Cholesterol (mM)	7.6 ± 2.2 *	34.2 ± 13.0	43.3 ± 18.5
Triglycerides (mM)	1.7 ± 0.6 *	6.0 ± 3.4	6.6 ± 4.8

Body composition, food intake and plasma lipids after 28 weeks of dietary treatment. BW: body weight; EI: energy intake; FI: food intake; eWAT: epididymal white adipose tissue (WAT); sWAT: subcutaneous WAT; mWAT mesenteric WAT. In the left column, data from the chow group are provided for reference. Data are presented as mean ± SD, * *p* < 0.05 compared to HFD. HFD: high-fat diet.

**Table 2:** Fatty acid concentrations in eWAT and the liver.

	eWAT			Liver		
Fatty Acids (Ug/Mg Tissue)	Chow	HFD	KrO	Chow	HFD	KrO
**Omega-3**						
Alpha-Linolenic (ALA) C18:3n3	11.0 *	4.8	5.5 *	0.7	0.7	0.9 *
Eicosapentaenoic (EPA) C20:5n3	0.3 *	0.1	1.5 *	0.3 *	0.2	2.5 *
Docosapentaenoic n3 C22:5n3	0.8	0.5	1.6 *	0.5 *	0.6	2.0 *
Docosahexaenoic (DHA) C22:6n3	1.9 *	1.1	4.0 *	3.7	3.9	7.6 *
**Omega-6**						
Linoleic (LA) C18:2n6	214.0 *	169.3	173.5	16.7 *	28.1	25.2
gamma-Linolenic C18:3n6	0.7	0.7	0.6	0.3 *	0.6	0.3 *
Eicosadienoic C20:2n6	1.1 *	2.5	2.4 *	0.3 *	0.7	0.6 *
Dihomo-g-linolenic C20:3n6	1.9 *	1.3	1.1 *	1.0 *	1.6	1.2 *
Arachidonic (ARA) C20:4n6	3.6 *	3.2	1.3 *	5.3 *	6.6	3.2 *
Docosatetraenoic C22:4n6	0.5 *	0.7	0.2 *	0.3 *	1.0	0.3 *
Docosapentaenoic n6 C22:5n6	0.3	0.3	0.1 *	0.1	0.5	0.0 *
**Cis-Monosaturated**						
Palmitoleic C16:1n7	79.8 *	32.8	34.1	5.0	5.9	6.8
Oleic C18:1n9	288.8 *	427.2	411.8	29.2 *	86.1	72.5
Eicosenoic C20:1n9	4.9 *	3.9	3.5 *	0.7 *	2.1	1.7 *
Nervonic C24:1n9	0.1 *	0.1	0.1	0.1	0.1	0.1 *
**Saturated**						
Myristic C14:0	7.7 *	5.5	6.4 *	0.5 *	1.0	0.8 *
Palmitic C16:0	152.6	133.4	130.3	21.4 *	46.4	38.6 *
Stearic C18:0	9.0 *	21.9	20.6	5.0 *	5.9	5.4 *
Arachidic C20:0	0.3 *	0.2	0.2	0.2	0.2	0.3
Behenic C22:0	0.1	0.0	0.0	0.1 *	0.0	0.1 *
Lignoceric C24:0	0.0 *	0.0	0.0	0.0 *	0.0	0.0 *
**Trans**						
Palmitelaidic C16:1n7t	0.8	0.7	0.9 *	0.1 *	0.1	0.2 *
Elaidic C18:1t	0.7 *	1.9	1.6 *	0.1 *	0.3	0.2 *
Linoelaidic C18:2n6t	2.0	1.6	1.5	0.3 *	0.5	0.3 *

Fatty acid concentrations determined in eWAT and liver tissue after 28 weeks of dietary treatment. The types of fatty acids are indicated in bold. In the left column, data from the chow group are provided for reference. Average fatty acid concentrations significantly higher compared to the HFD control group are shown in orange, and significantly lower concentrations are shown in green (* *p* < 0.05).

**Table 3:** Upstream regulator analysis in eWAT.

	HFD vs. Chow		KrO vs. HFD	
	Z-Score	*p*-Value	Z-Score	*p*-Value
**Proinflammatory**				
CCL2	3.4	0.000	−2.2	0.013
IL1B	6.7	0.000	−2.3	0.306
IL4	3.2	0.000	−0.1	0.016
IL6	3.5	0.000	−1.1	0.027
IL17A	2.5	0.000	−1.9	1.000
MIF	4.2	0.000	−1.9	1.000
TNF	9.5	0.000	−4.5	0.001
TRAP1	3.5	0.015	−2.4	0.001
TGFB1	4.7	0.000	−1.0	0.009
**Adipogenesis—Clonal Expansion**				
AP1	3.3	0.001	n/a	1.000
CEBPB	1.7	0.000	1.4	0.004
CEBPD	2.2	0.000	n/a	1.000
**Adipogenesis—Differentiation**				
PPARG	−4.9	0.000	5.0	0.000
RB1	−6.5	0.000	5.1	0.000
CEBPA	0.7	0.000	1.1	0.018
KLF4	−1.1	0.004	2.0	1.000
KLF5	−0.8	0.006	n/a	1.000
KLF15	n/a	1.000	3.0	0.000
SREBF1	−1.5	0.000	1.3	0.002
STAT5A	−1.2	0.000	2.8	0.004
STAT5B	−0.7	0.000	2.1	0.427
**Negative Regulators of Adipogenesis**				
KDM5A	4.6	0.000	−5.2	0.000
STK11	3.5	0.000	−4.6	0.000
KLF2	−1.2	0.000	n/a	1.000
GATA3	1.0	0.000	n/a	1.000
WNT5a	−0.5	0.000	n/a	1.000
**Metabolism-Related**				
PPARGC1A	−4.5	0.000	4.8	0.000
PPARGC1B	−2.6	0.018	2.6	0.001
ESRRA	−3.5	0.000	2.5	0.000
NRF1	−0.7	0.031	2.2	0.003
INSR	−3.4	0.000	3.9	0.000
IGF1R	−2.6	0.000	3.4	0.056
IRS1	−0.5	0.000	1.6	0.001

The activity of upstream regulators in eWAT was calculated based on gene expression changes of all downstream target genes. A Z-score < −2 indicates inhibition of the respective pathway (shown in orange) and Z > 2 indicates activation (shown in green). The *p*-value < 0.05 indicates significant enrichment of the target genes downstream of a regulator, i.e., that more downstream genes are affected than can be expected by chance. n/a indicates an insufficient number of differentially expressed genes to predict the activation state of an upstream regulator.

**Table 4:** Oxylipin analysis in eWAT.

	Chow	HFD	KrO
**Omega-3 Lipid Mediators**			
ALA/GLA	0.025	0.024	0.021
9-HoTrE	0.011	0.020	0.012
13-HoTrE	0.027 *	0.017	0.011
EPA	0.005	0.010	0.074 *
5-HEPE	0.001 *	0.001	0.011 *
12-HEPE	0.012	0.017	0.067 *
15-HEPE	0.003	0.005	0.026 *
18-HEPE	0.002 *	0.005	0.029 *
RvE2	ND	ND	ND
18R-RvE3	0.002	ND	ND
DPAn-3	0.003	0.006	0.018 *
DHA	0.019	0.042	0.099 *
4-HDHA	0.000 *	0.002	0.003 *
7-HDHA	0.001	0.002	0.004 *
10-HDHA	0.001 *	0.007	0.016 *
17-HDHA	0.002 *	0.007	0.013 *
PDX	0.001	0.002	0.004 *
14,15-diHETE	ND	0.002	0.004
14(S)-HDHA	0.007	0.013	0.024 *
19,20-DiHDPA	0.013 *	0.007	0.015 *
**Omega-6 Lipid Mediators**			
LA	0.059	0.060	0.074
9-HoDE	0.269 *	0.662	0.362 *
13-HoDE	0.506	0.959	0.569 *
DGLA	0.003 *	0.006	0.006
ARA	0.015 *	0.046	0.018 *
5-HETE	0.005 *	0.028	0.006 *
LXA4	0.001	0.002	ND
5-KETE	0.002 *	0.011	0.003 *
8-HETE	0.005 *	0.042	0.008 *
11-HETE	0.030 *	0.290	0.051 *
12-HETE	0.065	0.247	0.046 *
12-KETE	0.003	0.006	0.002
15-HETE	0.015 *	0.111	0.024 *
15-KETE	0.003 *	0.016	0.005 *
8S,15S-diHETE	0.001	0.002	0.000 *
17-OH-DH-HETE	0.001 *	0.005	0.001 *
PGD_2_	0.042 *	0.311	0.028 *
PGE_2_	0.057	1.226	0.052 *
PGF_2α_	0.006	0.034	0.004 *
PGJ_2_	ND	0.017	ND
8-iso- PGF_2α_	0.001 *	0.003	0.001 *
13,14 dihydro-15 keto- PGF_2α_	ND	0.009	ND
TxB_2_	0.012	0.051	0.005 *
6-tra-LTB_4_	0.001	0.003	0.001
6t,12 epi-LTB_4_	0.001	0.004	0.001
AdA	0.001 *	0.004	0.001 *
DPAn-6	0.002 *	0.011	0.002 *

Lipid mediator levels (area ratios relative to the class-specific internal standard) measured in eWAT tissue are expressed as mean area ratios. Area ratios significantly higher compared to the HFD control group are shown in orange, and significantly lower ratios are shown in green (* *p* < 0.05). In the left column, data from the chow group are provided for reference. ND = not detectable.

**Table 5:** Effect of KrO on adipokines, metabolic risk factors and transaminases.

	Chow	HFD	KrO
AUC leptin	32.3 ± 16.4 *	114.2 ± 15.4	100.4 ± 0.4 *
AUC adiponectin	18.5 ± 1.8 *	22.5 ± 2.5	25.19 ± 2.3 *
Glucose (mM)	7.2 ± 0.8	7.6 ± 0.9	6.9 ± 0.9 *
Insulin (ng/mL)	4.1 ± 3.9 *	20.4 ± 14.4	12.4 ± 7.1 *
HOMA-IR	34.1 ± 33.9 *	170.2 ± 116.3	92.2 ± 46.8 *
Free fatty acids (mM)	1.1 ± 0.2	1.3 ± 0.4	2.4 ± 1.0 *
ALT (U/l)	66.6 ± 21.5 *	391.9 ± 258.2	277.9 ± 170.7
AST (U/l)	17.8 ± 3.9 *	491.1 ± 497.2	264.2 ± 104.1

AUC: area under the curve, HOMA-IR: homeostatic model assessment for insulin resistance, ALT: alanine aminotransferase, AST: aspartate aminotransferase. In the left column, data from the chow group are shown for reference. Data are presented as mean ± SD, * *p* < 0.05 compared to HFD.

**Table 6:** Upstream regulator analysis in the liver.

	HFD vs. Chow		KrO vs. HFD	
Proinflammatory	Z-Score	*p*-Value	Z-Score	*p*-Value
CCL2	1.8	0.000	−2.0	0.171
CXCL12	4.2	0.000	−0.3	0.011
IL1B	7.9	0.000	−2.1	0.001
IFNA2	4.7	0.002	−1.9	0.036
IFNB1	2.7	0.000	−0.5	0.006
IFNG	6.1	0.000	−0.6	0.001
CSF1	5.1	0.000	−3.2	0.000
TNF	9.7	0.000	−1.1	0.000
NFKB1	4.8	0.000	−1.3	0.042
NFKBIA	3.2	0.000	0.2	0.029
RELA	4.9	0.000	−1.5	0.006
REL	3.2	0.000	−2.0	0.074
STAT1	6.4	0.000	−1.7	0.000
TICAM1	4.8	0.000	−2.6	0.068
**Metabolism-Related**				
ACOX1	−5.4	0.000	1.7	0.000
PPARGC1A	−2.7	0.000	1.7	0.004
SCAP	−3.2	0.000	−3.8	0.000
SREBF1	1.0	0.000	−2.6	0.000
SREBF2	−1.7	0.001	−3.2	0.000

The activity of an upstream regulator in the liver was calculated based on gene expression changes of all downstream target genes. A Z-score < −2 indicates inhibition of the respective pathway (shown in orange) and Z > 2 indicates activation (shown in green). The *p*-value < 0.05 indicates significant enrichment of the target genes downstream of a regulator, i.e., that more downstream genes are affected than can be expected by chance. n/a indicates an insufficient number of differentially expressed genes to predict the activation state of an upstream regulator.

**Table 7:** Oxylipin analysis in the liver.

	Chow	HFD	KrO
**Omega-3-Derived**			
ALA/GLA	0.208 *	0.304	0.143 *
9-HoTrE	0.070 *	0.016	0.022 *
13-HoTrE	0.036 *	0.015	0.013
EPA	0.283	0.267	1.765 *
5-HEPE	0.033 *	0.007	0.096 *
12-HEPE	0.559 *	0.172	1.077 *
15-HEPE	0.029 *	0.011	0.126 *
18-HEPE	0.052 *	0.012	0.169 *
RvE_2_	ND	ND	0.004
18R-RvE_3_	ND	ND	ND
DPAn-3	0.110 *	0.157	0.525 *
DHA	0.858 *	1.419	2.920 *
4-HDHA	0.037 *	0.015	0.039
7-HDHA	0.014	0.010	0.027 *
10-HDHA	0.066	0.049	0.122 *
17-HDHA	0.052	0.044	0.074 *
PDX	0.013	0.014	0.027 *
14,15-diHETE	0.062 *	0.028	0.206 *
14(S)-HDHA	0.123	0.126	0.155
19,20-DiHDPA	0.303 *	0.517	0.643 *
**Omega-6-Derived**			
LA	0.296	0.339	0.420 *
9-HoDE	1.567 *	0.935	0.746
13-HoDE	2.399 *	1.367	1.167
DGLA	0.095 *	0.189	0.215
ARA	0.372 *	0.658	0.348 *
5-HETE	0.150 *	0.083	0.037 *
LXA_4_	0.008	0.015	0.004
8-HETE	0.070	0.069	0.032 *
11-HETE	0.555	0.675	0.222 *
12-HETE	1.146	1.255	0.308 *
15-HETE	0.491	0.418	0.172 *
15-KETE	0.045	0.030	0.016 *
8S,15S-diHETE	0.005	0.006	0.002 *
17-OH-DH-HETE	0.016	0.019	0.004 *
PGD_2_	0.039 *	0.105	0.016 *
PGE_2_	0.068	0.103	0.023 *
PGF_2α_	0.041 *	0.095	0.012 *
PGJ_2_	ND	ND	ND
8-iso- PGF_2α_	0.003	0.004	0.002
13,14 dihydro-15 keto- PGF_2α_	ND	0.005	ND
TxB_2_	0.049 *	0.133	0.017 *
6-tra-LTB_4_	0.004	0.003	ND
6t,12epi-LTB_4_	0.006	0.004	ND
AdA	0.028 *	0.110	0.039 *
DPAn-6	0.073 *	0.323	0.047 *

Lipid mediator levels measured in liver tissue are expressed as mean area ratios. Area ratios significantly higher compared to the HFD control group are shown in orange, and ratios that are significantly lower are depicted in green (* *p* < 0.05). In the left column, data from the chow group are provided for reference. ND = not detectable.

## Data Availability

The gene expression data is publicly available on gene expression omnibus, data set GSE179396.

## Supplementary Materials

The following are available online at https://www.mdpi.com/article/10.3390/nu13082836/s1, Figure S1: PDX identification based on comparisons with a standard for PDX, Figure S2: RvE_2_ identification based on comparisons with a RvE_2_ standard, Table S1. Individually optimized LC-MS/MS parameters for each compound, Table S2. Fatty acid composition of dried blood spots and eWAT and liver tissue.
