# Supplementary material for: Krill Oil Treatment Increases Distinct PUFAs and Oxylipins in Adipose Tissue and Liver and Attenuates Obesity-Associated Inflammation via Direct and Indirect Mechanisms
Source: Nutrients. 2021 Aug 18;13(8):2836. doi: 10.3390/nu13082836 (PMC8401900; doi:10.3390/nu13082836)
Supplement: Supplementary file 1 [file nutrients-13-02836-s001.zip › nutrients-1300588-supplementary.pdf]

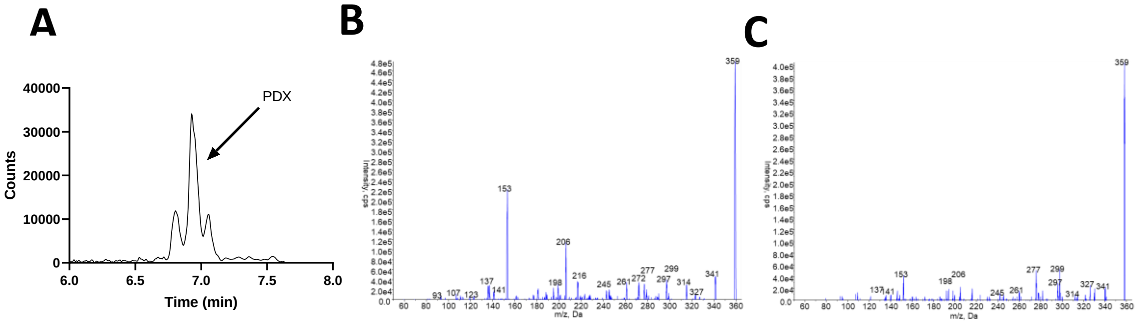

Supplemental figure S1. PDX identification based on comparisons with a standard for PDX. **A**) Chromatogram of PDX in a representative eWAT sample, **B**) PDX authentic standard material MS/MS spectrum, **C**) MS/MS spectrum obtained from representative WAT sample.

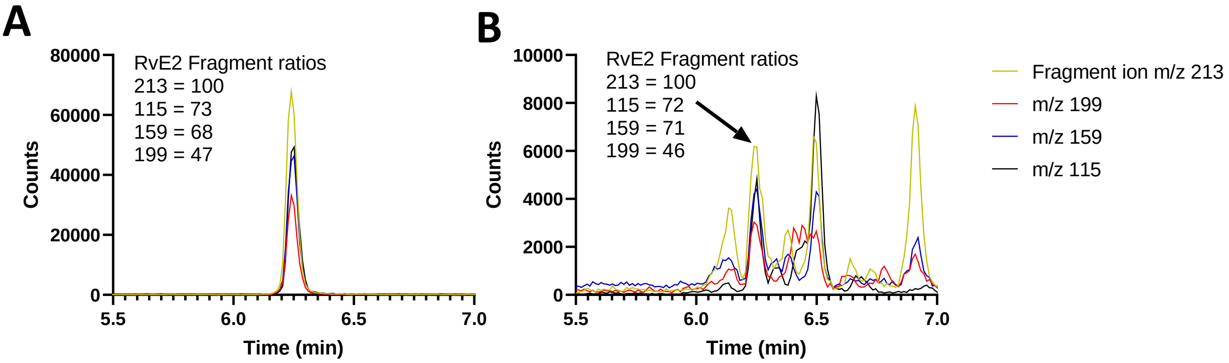

Supplemental figure S2. RvE_2 identification based on comparisons with a RvE_2 standard. The graphs show characteristic fragment ions of RvE2 with a dedicated product ion scan LC-MS/MS: **A**) 1ng/ml authentic standard material, **B**) RvE2 fragments from a representative liver sample.

Supplemental Table S1. Individually optimized LC-MS/MS parameters for each compound.

ID	m/z in Q1	m/z in Q3	Time (min)	DP (volts)	EP (volts)	CE (volts)	CXP (volts)
LXA4	351.1	114.8	5.5	-40	-10	-20	-11
5-HETE	319.1	115.0	8.0	-65	-10	-18	-11
8-HETE	319.1	154.9	7.9	-70	-10	-20	-19
11-HETE	319.1	167.0	7.9	-70	-10	-22	-15
12-HETE	319.1	179.0	7.9	-65	-10	-20	-23
15-HETE	319.1	219.1	7.8	-55	-10	-18	-9
15-HEPE	317.1	219.0	7.5	-65	-10	-18	-19
18-HEPE	317.1	259.0	7.4	-5	-10	-16	-7
LXB4	351.1	220.9	5.1	-60	-10	-22	-13
Leukotriene B4	335.1	195.0	6.9	-65	-10	-22	-21
17-HDHA	343.1	245.0	7.9	-65	-10	-16	-15
14,15-diHETE	335.1	207.0	7.0	-65	-10	-24	-21
19,20-DiHDPA	361.1	273.0	7.4	-55	-10	-22	-15
RvE1	349.1	195.0	3.8	-95	-10	-22	-13
RvE2	333.1	114.9	6.1	-35	-10	-18	-15
18S-RvE3	333.1	245.2	6.7	-25	-10	-16	-17
18R-RvE3	333.1	245.0	7.1	-55	-10	-18	-23
TxB2	369.1	169.0	4.6	-55	-10	-24	-15
6-trans-LTB4	335.1	194.9	6.7	-105	-10	-22	-11

20-OH LTB4	351.1	195.0	4.0	-60	-10	-24	-17
PGD2	351.1	233.0	5.0	-30	-10	-16	-13
PDX	359.1	153.0	6.8	-70	-10	-22	-9
PD1	359.1	153.0	6.9	-70	-10	-22	-9
MaR1_2	359.2	250.2	7.0	-65	-10	-20	-13
LTE4	438.1	333.1	7.0	-55	-10	-26	-15
8S,15S-diHETE	335.1	207.9	6.7	-55	-10	-22	-17
LTD4	495.1	177.0	6.7	-70	-10	-28	-19
Leukotriene B4 d4	339.1	196.9	6.9	-70	-10	-22	-19
15-HETE d8	327.2	226.0	7.8	-85	-10	-18	-11
PGE2-d4	355.1	193.0	4.9	-50	-10	-26	-17
7,17-DiHDPA	361.1	198.9	7.0	-45	-10	-26	-23
RvD1	375.1	215.0	5.6	-50	-10	-26	-11
RvD2	375.1	277.1	5.3	-60	-10	-18	-15
6t,12epi-LTB4	335.1	194.9	6.8	-80	-10	-22	-25
PGF2a	353.1	193.0	5.2	-80	-10	-34	-11
PGE2_2	351.2	271.1	4.9	-50	-10	-22	-21
17-OH-DH-HETE	347.1	247.0	8.2	-110	-10	-22	-27
13-HoTrE	293.0	195.0	7.4	-45	-10	-24	-19
13-HoDE	295.0	194.9	7.7	-110	-10	-24	-21
7S-MaR1	359.1	249.9	6.6	-20	-10	-20	-19
15-Keto-PGE2	349.0	234.9	4.5	-65	-10	-20	-13
13,14dihydro-15keto-PGF2a	353.1	195.0	5.4	-110	-10	-32	-11
8-iso-PGE2	351.1	271.0	4.6	-5	-10	-24	-19
8-iso-PGF2a	353.1	193.0	4.5	-135	-10	-34	-11
9-HoTrE	293.0	170.9	7.4	-75	-10	-20	-15
9-HoDE	295.0	171.0	7.7	-130	-10	-22	-7
AA	303.0	205.1	8.8	-155	-10	-20	-11
DHA	327.1	229.2	8.8	-115	-10	-18	-11
EPA	301.0	202.9	8.6	-125	-10	-18	-21
AdA	331.1	233.0	9.1	-130	-10	-22	-11
DPAn-3	329.1	231.1	9.0	-50	-10	-20	-17
DHAd5	332.0	288.1	8.8	-75	-10	-16	-13
LA	279.0	261.0	8.8	-115	-10	-28	-13
ALA/GLA	277.0	233.0	8.6	-90	-10	-22	-29
PGJ2	333.0	271.0	6.1	-30	-10	-22	-17
5,15-diHETE	335.0	173.1	6.8	-55	-10	-20	-11
10-HDHA	343.1	153.0	7.9	-25	-10	-20	-15
7-HDHA	343.1	141.1	8.0	-85	-10	-18	-23
14(15)EET	319.0	218.9	8.1	-5	-10	-16	-55
11(12)EET	318.9	166.9	8.2	-90	-10	-18	-19
8(9)EET	319.0	154.9	8.2	-60	-10	-18	-13
15Deoxy PGJ2	315.0	203.0	7.3	-50	-10	-28	-19
20-HETE_2	319.0	289.1	7.7	-70	-10	-24	-15
4-HDHA	343.1	101.0	8.2	-50	-10	-18	-9
14(S)-HDHA	343.1	204.9	8.0	-60	-10	-18	-27
5-HEPE	317.0	114.9	7.7	-55	-10	-18	-11
12-HEPE	317.0	179.0	7.6	-60	-10	-18	-17
5-KETE	317.0	203.1	8.1	-70	-10	-24	-11
12-KETE	317.0	153.0	7.9	-60	-10	-22	-9
15-KETE	317.0	113.0	7.8	-10	-10	-22	-5

19(20)-EpDPA	343.1	281.1	8.1	-70	-10	-16	-11
DGLA	305.1	261.2	9.0	-85	-10	-22	-13
8-iso-PGF2alpha-d4	357.3	197.0	4.5	-110	-10	-34	-20
14(15)-EET-d11	330.2	219.0	8.1	-85	-10	-15	-15

Supplemental Table S2. Fatty acid composition of dried blood spots and eWAT and liver tissue.

	**Dried blood spots**			**eWAT**			**Liver**		
**Fatty acids (% of all FA measured)**	**chow**	**HFD**	**KrO**	**chow**	**HFD**	**KrO**	**chow**	**HFD**	**KrO**
**Omega-3**									
**alpha-Linolenic C18:3n3**	0.2%*	0.2%	0.3%*	1.5%*	0.6%	0.7%*	0.8%*	0.4%	0.5%*
**Eicosapentaenoic (EPA) C20:5n3**	0.6%*	0.3%	6.6%*	0.1%*	0.0%	0.2%*	0.4%*	0.1%	1.6%*
**Docosapentaenoic n3C22:5n3**	0.6%*	0.3%	0.8%*	0.1%*	0.1%	0.2%*	0.6%*	0.3%	1.2%*
**Docosahexaenoic (DHA) C22:6n3**	5.7%*	4.5%	6.7%*	0.3%*	0.1%	0.5%*	4.5%*	2.0%	4.7%*
**Omega-6**									
**Linoleic C18:2n6**	18.4%	18.1%	18.7%	27.1%*	20.8%	21.6%*	18.6%*	14.7%	15.0%
**gamma-Linolenic C18:3n6**	0.3%	0.3%	0.2%*	0.1%*	0.1%	0.1%	0.3%	0.3%	0.2%*
**Eicosadienoic C20:2n6**	0.2%*	0.3%	0.4%*	0.1%*	0.3%	0.3%	0.3%*	0.4%	0.3%*
**Dihomo-g-linolenic C20:3n6**	1.4%	1.3%	1.3%	0.3%*	0.2%	0.1%*	1.1%*	0.9%	0.7%*
**Arachidonic (ARA) C20:4n6**	18.9%*	20.7%	8.4%*	0.6%*	0.4%	0.2%*	6.6%*	3.5%	2.0%*
**Docosatetraenoic C22:4n6**	0.9%*	0.6%	0.1%*	0.1%*	0.1%	0.0%*	0.3%*	0.5%	0.2%*
**Docosapentaenoic n6C22:5n6**	0.5%	0.4%	0.0%*	0.0%	0.0%	0.0%*	0.2%*	0.3%	0.0%*
**Cis-Monosaturated**									
**Palmitoleic C16:1n7**	2.2%*	1.6%	3.1%*	10.4%	4.0%*	4.3%*	5.1%*	3.0%	3.9%*
**Oleic C18:1n9**	15.6%*	20.2%	23.0%*	36.5%	52.6%*	51.3%*	29.7%*	44.4%	41.8%*
**Eicosenoic C20:1n9**	0.4%	0.4%	0.4%	0.6%	0.5%*	0.4%*	0.7%	1.1%	1.0%*
**Nervonic C24:1n9**	0.5%*	0.2%	0.3%*	0.0%	0.0%*	0.0%	0.1%*	0.0%	0.1%*
**Saturated**									
**Myristic C14:0**	0.2%	0.1%	0.2%*	1.0%	0.7%*	0.8%*	0.5%	0.5%	0.5%*
**Palmitic C16:0**	21.8%*	18.7%	19.3%	19.6%	16.4%*	16.2%	23.5%	23.9%	22.6%*
**Stearic C18:0**	10.3%	10.7%	9.0%*	1.2%	2.7%*	2.7%	6.1%*	3.1%	3.5%
**Arachidic C20:0**	0.2%*	0.2%	0.2%	0.0%	0.0%*	0.0%	0.3%*	0.1%	0.2%*
**Behenic C22:0**	0.5%*	0.2%	0.2%	0.0%	0.0%	0.0%	0.1%*	0.0%	0.0%*
**Lignoceric C24:0**	0.5%*	0.2%	0.2%	0.0%	0.0%*	0.0%	0.1%*	0.0%	0.0%*
**Trans**									
**Palmitelaidic C16:1n7t**	0.1%	0.1%	0.1%*	0.1%	0.1%*	0.1%*	0.1%	0.1%	0.1%*
**Elaidic C18:1t**	0.2%*	0.2%	0.2%	0.1%	0.2%*	0.2%*	0.1%*	0.1%	0.1%*
**Linoelaidic C18:2n6t**	0.2%	0.2%	0.2%*	0.3%	0.2%*	0.2%	0.3%*	0.3%	0.2%*

Average fatty acid percentages after 28 weeks of dietary treatment are shown (percentage of totally identified fatty acids) with *p<0.05 compared to HFD. Data of the chow group was provided for reference.
